# Adaptive Stochastic Resonance-Based Processing of Weak Magnetic Slippage Signals of Bearings

**DOI:** 10.3390/e24020147

**Published:** 2022-01-19

**Authors:** Jianpeng Ma, Chengwei Li, Guangzhu Zhang

**Affiliations:** 1 School of Instrumentation Science and Engineering, Harbin Institute of Technology, Harbin 150001, China; 18B901017@stu.hit.edu.cn; 2 Songsim Global Campus, Undergraduate College, The Catholic University of Korea, Bucheon-si 14662, Korea; zhangks@catholic.ac.kr

**Keywords:** bearing weak magnetic signal, stochastic resonance, adaptive algorithm, feature extraction, slip rate

## Abstract

Slip is one of the most common forms of failure in aviation bearings, and it can pose a great threat to the stable operation of aviation bearings. Bearing cage speed monitoring methods based on weak magnetic detection can achieve nondestructive measurements. However, the method suffers from solid signal background noise due to the high sensitivity of the sensor. Therefore, in this paper, an adaptive stochastic resonance algorithm was proposed in response to the characteristics of the weak magnetic detection signal and the problem of solid noise. In addition, by adaptively adjusting the coefficients of the stochastic resonance system—by an improved moth flame optimization algorithm—the drawback in which the stochastic resonance method required artificially set parameters for extracting the feature frequencies of the weak magnetic signals was solved. In this process, we used parameters, such as general refined composite multi-scale sample entropy, as the adaptation function of the optimization algorithm. In the end, simulation and experimental outcomes verified the efficacy of the approach put forward.

## 1. Introduction

In the case of high-speed operation, an aero-engine main bearing rolling body, driven by inertial force, cannot maintain a pure rolling state with the inner ring. The difference between the linear velocity of the contact point of the rolling body and the inner ring will lead to relative sliding between the rolling body and the outer ring (i.e., the slip phenomenon). This phenomenon is widespread at high speeds and during light load operation. Reduction in cage speed is a common form of slip, and is therefore referred to as cage slip [1]. Severe slippage can lead to surface spalling and other forms of failure, posing a significant threat to stable engine operation [2]. With the development of high-speed aero engines and lightweight spindles, slippage problems are becoming more common. Therefore, the calculation of bearing slip rate is of great importance to the operating accuracy and service life of aero engines.

Traditional measurement means have many problems [3]; in fact, a recently proposed method based on weak magnetic detection bridged the gap of previous studies [4]. However, this method, based on weak magnetic detection, created a new problem: excessive signal background noise. The mechanism of the weak magnetic examination method works such that the inner ring and rolling body of a bearing are magnetically conductive and are therefore magnetized by the influence of geomagnetism. During the rotation of the bearing, the geomagnetic field around it changes weakly. This weak change can be detected by the weak magnetic sensor [5]. However, due to the high sensitivity of the weak magnetic sensor, the signal acquisition is subject to more interference, resulting in intense signal noise. The weak rotational frequency can be hidden by the noise. In this study, an adaptive stochastic resonance algorithm was proposed to solve the feature extraction problem of the weak magnetic detection method under bearing speed measurement and improve its engineering.

The difficulties of weak magnetic signal extraction, compared with the traditional vibration weak signal, can be summarized as follows: (1) when processing a traditional vibration weak signal, only one characteristic frequency is needed to determine the early fault type. However, weak magnetic signal processing needs to extract three characteristic frequencies simultaneously: cage rotation frequency, inner ring rotation frequency, and rolling body rotation frequency (which also contains the sum of weak signals with multiple multiples and different characteristic frequencies). Meanwhile, there is a correlation between the rotational frequency of the rolling body and the rotational frequency of the cage. Essentially, the rotational frequency of the rolling body is equivalent to the goods of the rotational frequency of the cage and the number of the rolling body. (2) In the traditional vibration weak signal, the characteristic frequency of bearing failure is a fixed value. It is only related to the parameters and rotational speed of the bearing and has nothing to do with the severity of the failure or the operation time. In contrast, in the weak magnetic signal method, the cage rotation frequency is a variable characteristic quantity that varies with time, and the monitored value is less than the theoretical value. Therefore, to settle the issue of weak magnetic examination methods in bearing slip measurement, there was an urgent need for a signal processing method capable of extracting the feature frequency of the weak magnetic signal.

At the time of this work, the study of weak magnetic signals for aerospace bearings was available only in the CEEMDAN algorithm [4], but the filtering effect was unsatisfactory. The commonly used weak signal processing algorithms are: wavelet transform [6], empirical modal decomposition [7], and singular value decomposition [8]. However, these methods have the following problems: (1) the valuable signal is suppressed along with the noise in the process of filtering the weak signal, (2) none of these methods can strengthen the signal-to-noise proportion of the system, so the output signal-to-noise proportion of the system decreases with the growth of the background noise, and (3) it is hard to achieve the required level of detection sensitivity under intense background noise.

All of the aforementioned methods have been understood to have certain limitations when processing weak signals. However, the stochastic resonance theory, put forward by Benzi et al. [9] in describing the phenomenon of periodic alternation between the Earth’s glacial and warm climate periods, provided a new idea to solve the weak signal extraction, and its theory was opposed to the above filtering method idea [9]. Its core lay in using noise, rather than filtering it, to transform part of the noise energy in nonlinear systems to the input signal energy of weak signals, thus enhancing the system’s output response. This theory suggested a new method for extracting valuable signals in a strong noise background [10].

Scholars have begun to use nonlinear stochastic resonance techniques to extract and detect weak signals more widely [11,12,13]. Early on in the research, scholars enhanced the weak periodic input signal and increased the output signal-to-noise ratio of the system by adding different intensities of noise to the stochastic resonance system. Later, scholars discovered that stochastic resonance could be generated by modifying the system parameters without changing the noise intensity (i.e., parameter-modulated stochastic resonance). Xu and Duan [14] showed that, if the system noise intensity exceeded the intensity scope that makes stochastic resonance occur, optimal adjustment of the system parameters could acquire the optimal system response rate and make the system output lie in the resonance area. Li et al. [15] extracted fault features of boring and milling machine tools by introducing an adaptive stochastic resonance signal processing technique and obtained superior system output performance over the traditional fast Fourier transform mechanism. Qiao et al. [16] investigated the effects of pleasing potential asymmetry on detecting weak periodic signals of bearing faults in a bistable stochastic resonance system and found that the control system potential function could control the system. Qiao et al. [17] also overcame the drawback of inherent output saturation in the classical bistatic stochastic resonance method by putting forward a segmented bistatic potential model. Zhang et al. [18] optimized the bistatic stochastic resonance parameters and adjusted the parameters adaptively with the gray wolf optimization algorithm. Li et al. [19] proposed a new adaptive stochastic resonance model on the basis of coupled bistable systems. Yaguo Lei et al. [20] proposed an underdamped multi-stable stochastic resonance system model for weak signals, outperforming EEMD and conventional overdamped bistable stochastic resonance methods. Zhang et al. [21] studied a coupled fractional-order linear stochastic resonance model with underdamped nonlinear frequency rise and fall of particles. Through the summary of the above stochastic resonance method studies, it was found that there have been few studies for coupled underdamped multi-stable stochastic resonance. Considering that the underdamped potential function could be regarded as the property of a quadratic filter, the coupling coefficient was introduced, to take into account the impact of the mutual effect between subsystems on the system output. Consequently, it was essential for the extraction of characteristic frequencies of weak magnetic signals under solid noise.

In this work, a method for adaptive extraction of characteristic frequencies in weak magnetic signals was proposed. An underdamped tri-stable stochastic resonant system model was proposed first. The proposed stochastic resonant system model behaved as a band-pass filter, enhancing the periodic features of the target signal and attenuating the low and high-frequency disturbances. Second, an enhanced moth flame optimization algorithm was proposed as a solution for the adaptive problem. Although some adaptive stochastic resonance methods have been proposed [22,23,24], these improvement algorithms (i.e., genetic algorithms, particle swarm improvement, and grid search methods) still suffer from the problem of limited global optimization capability [25,26,27,28]. Considering that the performance and efficiency of the adaptive stochastic resonance method will be directly affected by the global optimization capability of an optimization algorithm, the moth flame improvement algorithm [29]—which is simple, flexible, robust, and close to the global optimum—was selected and improved upon. Moreover, the model’s effectiveness (with the adaptive underdamped tri-stable stochastic resonant system proposed in this paper) in extracting weak magnetic signals was verified through simulated and experimental weak magnetic signals.

The remaining sections of the paper are arranged as below. Section 2 introduces associated works, such as the traditional theory of stochastic resonant systems and the moth flame improvement algorithm. Section 3 introduces the method put forward in this paper. Section 4 verifies the efficacy of the method put forward in this paper in extracting weak signals using simulated signals. It also introduces the experimental platform and the experimental procedure through which the efficacy of the approach put forward in this paper is demonstrated. Finally, conclusions are provided in Section 5.

## 2. Related Works

### 2.1. Stochastic Resonance System

Stochastic resonance is a nonlinear physical phenomenon in which the noise enhances the output response of a system under the synergistic role of the driving force (input signal), the random force (noise), and the nonlinear system [30]. Normally, noise is considered a source of influence on the system performance during signal processing of linear systems and causes troubles in the extraction and detection of useful information. However, the stochastic resonance-based mechanism, which scholars have extensively studied in recent years, suggests that the weak input signal of the system in some specific nonlinear systems can be enhanced with noise. The three elements of the stochastic resonance phenomenon occurring are: the weak input signal, noise, and nonlinear signal processing system. The synergy between these three will transfer disordered noise energy into ordered signal energy so that the nonlinear system output response is the enhanced weak useful signal. To date, as stochastic resonance is explored continuously, multi-stable stochastic resonance has caused a lot of concerns from various scholars. It is well understood that the theory of stochastic resonance was illustrated by the nonlinear Langevin equation from the view of Brownian particle motion. Hence, the equation was extended to multi-stable systems by this section, and the action of stochastic forces on the systems was introduced briefly.

The kinetic equations of Brownian particles are used to illustrate the laws of motion of Brownian particles in liquids, and the Langevin equation was established on this basis. The macroscopic equation for the motion of a Brownian particle of mass in a liquid m is [31]:

(1) $$m\dot{v}=-\alpha v$$

where $\dot{v}$ means the speed of motion of the Brownian particles in the liquid and −αv stands for the viscous force. In the case of a large mass of the Brownian particle, except the macroscopic viscous force, the Brownian particle will be subject to the force of the liquid molecules in all directions for irregular motion. In the case of smaller mass of the Brownian particle, the amplitude of the motion will be larger; thus, the force of the collision of the haphazard molecules is added to Equation (1) [32]:

(2) mv=−αv+F(t)

where F(t) refers to the total force provided by colliding liquid molecules with Brownian particles. Both sides of Equation (2) by the mass is divided at the same time yields

(3) $$\dot{v}+rv=\Gamma \left(t\right)$$

where $r=\frac{\alpha }{m}\Gamma \left(t\right)=\frac{F\left(t\right)}{m}$ are the damping parameter per unit mass and the molecular collisional up and downforce, respectively, Γ(t) is the Rangzwan force, and Equation (3) is known as the Rangzwan equation. Assuming that the particle t=0 has the velocity, position at, then will be $v=\dot{x}$ brought as in Equation (3), so get

(4) $$\ddot{x}+r\dot{x}=\Gamma \left(t\right)$$

Since the Brownian particle is still subject to outer forces, the Rangzwan equation with the action of external field forces becomes [33]

(5) $$\ddot{x}+r\dot{x}=f\left(x\right)+\Gamma \left(t\right)$$

where f(x) means the outer force applied to the mean unit mass Brownian particle. If overdamping, the left-hand side of Equation (5) means the damping term, exerting the main effects, so as to reduce the inertia term, and such that r=1 Equation (5) to

(6) $$\dot{x}=f(x)+\Gamma \left(t\right)$$

If the outer force on a mean unit mass Brownian particle f(x) is a nonlinear multi-stable potential function, the equation displayed in Equation (6) stands for the nonlinear Langevin equation. Because of no fixed solution of the nonlinear Langevin equation researchers have turned to the evolution law of the Langevin equation. Let ρ(x,t) be x the distribution function, whose Markov process is described as

(7) $$P\left(x_{n},t_{n}|x_{n-1},t_{n-1};\ldots ;x_{1},t_{1}\right)=P\left(x_{n},t_{n}|x_{n-1},t_{n-1}\right)$$

Its distribution function is

(8) $$\rho \left(x,t+\tau \right)=\int P\left(x,t+\tau |x^{\prime },t\right)\rho \left(x^{\prime },t\right)dx^{\prime }$$

Expanding the above equation yields

(9) $$\frac{\partial \rho \left(x,t\right)}{\partial t}=\left\{\sum_{n=1}^{\infty }{\left(-\frac{\partial }{\partial x}\right)}^{n}\underset{\tau \to 0}{\lim}\frac{M_{n}\left(x,t,\tau \right)}{n!\tau }\right\}\rho \left(x,t\right)$$

By Equation (9), if the solution of the function ρ(x,t) is to be obtained, the leap moment $M_{n}\left(x,t,\tau \right)$ needs to be known. Equation (6) at the moment of time, the t+τ center distance of each order centered at x

(10) $$M_{n}\left(x,t,\tau \right)=\langle {\left[x\left(t+\tau \right)-x\right]}^{n}\rangle$$

(11) $$x\left(t+\tau \right)-x=\int_{t}^{t+\tau }\left[f\left(x\left(t^{\prime }\right),t^{\prime }\right)\right]dt^{\prime }$$

The Focke–Planck equation (FPE) can be obtained by substituting the above equation into Equation (9) as

(12) $$\frac{\partial \rho \left(x,t\right)}{\partial t}=-\frac{\partial }{\partial x}\left\{\left[f\left(x,t\right)+D\eta \left(x,t\right)\eta^{\prime }\left(x,t\right)\right]\rho \left(x,t\right)\right\}+D\frac{\partial^{2}}{\partial x^{2}}\left[\eta^{2}\left(x,t\right)\rho \left(x,t\right)\right]$$

When the external force on the particle in the nonlinear Rangzevan equations Γ(t)=ξ(t) is substituted into Equation (6), we get:

(13) $$\dot{x}=-U^{\prime }\left(x\right)+s(t)+\xi \left(t\right)$$

where U(x) means a category of nonlinear multi-stability hidden functions according to Equation (13), where b and c are the system coefficients and are all larger than 0.

(14) $$U\left(x\right)=\frac{a}{2}x^{2}-\frac{1+a}{4b}x^{4}+\frac{c}{6}x^{6}$$

Assuming that the system input signal is, substituting Equation (14) into Equation (13) yields

(15) $$\dot{x}=-ax+\frac{1+a}{b}x^{3}-cx^{5}+s(t)+\xi \left(t\right)$$

where s(t) refers to the input signal of various frequencies superimposed, standing for the outer force according to Equation (16):

(16) $$s\left(t\right)=\sum_{i=1}^{n}A_{i}\sin(2\pi f_{i}t),i=1,2,\ldots ,n$$

where $A_{i}$ means the amplitude of the first signal, i$f_{i}$ represents the frequency of the first i signal, and n represents the number of input signals. Assuming ξ(t) that the statistical features of the input signal are displayed in Equation (17), where D means the noise intensity and $t^{\prime }$ is the time delay about t:

(17) $$\langle \xi \left(t\right)\rangle =0,\langle \xi \left(t\right)\xi \left(t^{\prime }\right)\rangle =2D\delta \left(t-t^{\prime }\right)$$

According to Equation (17), the power spectrum is obtained by Fourier expansion of ξ(t)

(18) $$S\left(\omega \right)=\int e^{-i\omega \tau }2D\delta \left(\tau \right)d\tau =2D$$

It can be seen from Equation (18) that its power spectrum is uncorrelated S(ω) with ω the spectrum, i.e., the spectrum is white, so the Rangzwan force in Equation (17), with a time-correlated function of ξ(t) Rangzwan, is called white noise. Meanwhile, the nonlinear Rangzwan equation can be written as

(19) $$\dot{x}=-ax+\frac{1+a}{b}x^{3}-cx^{5}+s(t)+D\xi \left(t\right)$$

The above equation is a class of nonlinear Langevin equations with three steady-status points, whose essence is the periodic leap of a particle in the hidden well of a nonlinear system with the effect of all forces.

Equation (19) describes the multi-steady SR system with three steady-status solutions (minimal value points) and two unstable solutions (maximal value points) in the case of the system in the equilibrium stable-state, as shown in Equations (20) and (21), respectively.

(20) $$\{\begin{array}{c} -x_{2}=-\sqrt{\frac{1}{2c}\left(\frac{1+a}{b}+\sqrt{{\left(\frac{1+a}{b}\right)}^{2}-4ac}\right)}\\ x_{0}=0\\ x_{2}=\sqrt{\frac{1}{2c}\left(\frac{1+a}{b}+\sqrt{{\left(\frac{1+a}{b}\right)}^{2}-4ac}\right)} \end{array}$$

(21) $$\{\begin{array}{c} -x_{1}=-\sqrt{\frac{1}{2c}\left(\frac{1+a}{b}-\sqrt{{\left(\frac{1+a}{b}\right)}^{2}-4ac}\right)}\\ x_{1}=\sqrt{\frac{1}{2c}\left(\frac{1+a}{b}-\sqrt{{\left(\frac{1+a}{b}\right)}^{2}-4ac}\right)} \end{array}$$

The corresponding intermediate hidden well depths $\Delta U_{M}$ and the depths of the hidden wells on both sides $\Delta U_{L,R}$ are

(22) $$\Delta U_{M}=\frac{1}{24c^{2}}\left[{\left({\left(\frac{1+a}{b}\right)}^{2}-4ac\right)}^{3/2}-\frac{1+a}{b}\left({\left(\frac{1+a}{b}\right)}^{2}-6ac\right)\right]$$

(23) $$\Delta U_{L,R}=\frac{1}{12c^{2}}{\left[{\left(\frac{1+a}{b}\right)}^{2}-4ac\right]}^{3/2}$$

### 2.2. Moth Flame Optimization Algorithm

Seyedali Mirjalili, inspired by the lateral localization navigation mechanism of moths, mathematically modeled the spiral flight behavior of moths to flames in 2015 and solved optimization problems in different domains [34] by proposing a moth flame optimization algorithm. In MFO, moths are searching for the optimal individual in the search space, and the optimal position that the current moth population can find is saved and assigned to the flame. Each moth continuously adjusts its flight trajectory toward the optimal global solution using the corresponding flame as a guidance for the search. The MFO algorithm is described in detail below.

(1) Population initialization

The MFO algorithm assumes that moths are candidate solutions and the variable of the issue is the position of the moths in the search space. The moth population is displayed in the matrix as below.

(24) $$M=\left[\begin{array}{cccc} M_{1,1} & M_{1,2} & \ldots & M_{1,d}\\ M_{2,1} & M_{2,2} & \ldots & M_{2,d}\\ \vdots & \vdots & \vdots & \vdots \\ M_{n,1} & M_{n,2} & \ldots & M_{n,d} \end{array}\right]$$

where n denotes the number of moths and d denotes the number of variables.

For all moths, the related fitness values are stored with an array OM, as shown in Equation (25).

(25) $$OM=\left[\begin{array}{c} OM_{1}\\ OM_{2}\\ \vdots \\ OM_{n} \end{array}\right]$$

Another core ingredient in the MFO algorithm is the flame, and the flame position is a matrix of variables of the same dimension as the moth position.

(26) $$F=\left[\begin{array}{cccc} F_{1,1} & F_{1,2} & \ldots & F_{1,d}\\ F_{2,1} & F_{2,2} & \ldots & F_{2,d}\\ \vdots & \vdots & \vdots & \vdots \\ F_{n,1} & F_{n,2} & \ldots & F_{n,d} \end{array}\right]$$

Again, the array OF is used for all flames to store the corresponding fitness values, as shown in Equation (27).

(27) $$OF=\left[\begin{array}{c} OF_{1}\\ OF_{2}\\ \vdots \\ OF_{n} \end{array}\right]$$

(2) Position update process

The MFO algorithm assigns a specific flame to each moth using a logarithmic spiral function to update the moth’s position with the following equation.

(28) $$M_{i}=S\left(M_{i},F_{j}\right)=D_{i}\cdot e^{bt}\cdot \cos\left(2\pi t\right)+F_{j}$$

where $D_{i}=\left|F_{j}-M_{i}\right|$ means the distance between the moth $M_{i}$ and the flame $F_{j}$, b refers to a constant associated with the shape of the spiral, the random number denotes the closest place to the flame and t=1 is the farthest position from the flame. In the optimization process, to further enhance the exploitation capability, it is predicted that a random number decreases linearly r from −1 to −2.

There are several flames at the initial iteration stage, and the MFO algorithm adaptively reduces the number of flames until the last optimal flame is retained (see Equation (29).

(29) $$flame\_no=round(n-l\frac{n-1}{T})$$

where l stands for the number of current iterations, n means the most number of flames, and T is the most number of iterations.

The adaptive reduction of the number of flames during the iterations balances the digging and exploitation capabilities of the algorithm. As the iteration progresses, the number of flames grows smaller and smaller, and the number of moths becomes relatively redundant—so that the redundant moths update their positions around the first flame, flame_no.

Compared with other metaheuristic algorithms, the MFO algorithm saves the optimal solution that the moth population can find in each iteration and assigns it to the flame. The unique one-to-one update mechanism of the algorithm greatly increases the digging of the search space and decreases the possibility of the algorithm falling into a local optimum. The adaptive reduction of the number of inferior solutions improves optimization efficiency, and these excellent features give the algorithm strong global search capabilities for optimal performance and robustness. The basic steps of the moth flame optimization algorithm are shown in Algorithm 1.
**Algorithm 1.** MFO pseudo code1: initialization parameters: population size n, number of dimensions, the maximum number of iterations.2: random initialization of moth positions in the search space M.3: While (l<=T)4: Calculate the fitness of each moth OM.5: Calculate the number of flames using Equation (29).6: If the current iteration number l=1, then F=sort(M) update the flame population according to.7: otherwise update the flame population according to,8: recording the first flame as the optimal individual.9: for to i=1 do n10: Update r,11: Update the moth position using Equation (28).12: Determine whether the location of individual moths exceeds the upper and lower limits of the search space.13: re-initialize the position in the search space if it is out of bounds.14: end for15: end while16: Output optimal solution

## 3. Proposed Method

### 3.1. Improved Multi-Stable Stochastic Resonance Model

The resonance phenomenon is essential that the particle oscillates within the hidden under the integrated excitation of the hidden, the periodic force, and the noise force. Among them, the gradient (U(x) of the first-order derivative) of the potential curve generates the hidden force. Given that the periodic and noise forces are not always correctable in real situations, the efficiency of particle oscillations can be significantly influenced by the potential; for instance, suppose a too wide potential. In this case, the particle cannot realize the hidden all within one periodic excitation cycle (always required to provide maximum restoring force). Thus, the restoring force does not help to enhance the periodic motion of the particle. Conversely, in the case of a too narrow potential wall, the particle may not have a chance to arrive at the desired destination forward in one excitation cycle (because the hidden wall may have compelled the particle to move backward prematurely). Thus, the potential does not strengthen the periodic motion and also induces unexpected noise. Likewise, steep potential walls result in strong recovery forces, in turn leading to rapid particle bouncing. However, when the recovery velocity is too high, it may cancel because the periodic oscillations cannot keep up with the recovery velocity. Conversely, in the case of too falt potential wall, enough acceleration may not be provided to strengthen the periodic oscillations. Therefore, if the potential is within the best conditions to match the periodic force, the periodic and noise forces have the opportunity to amplify the particle oscillations. Then, the periodic signal can be strengthened. On the basis of the above analysis, the potential model needs to be well-tuned to enhance the periodic signal to the extreme.

On the basis of the features of the weak magnetic signal of the bearing, this paper proposed using an underdamped model instead of the conventional overdamped tri-stable model. Similarly, the Rangzwan equation for the underdamped stochastic resonance method was found in [20]:

(30) $$\frac{d^{2}x}{dt^{2}}=-\frac{dU\left(x\right)}{dx}-\beta \frac{dx}{dt}+A\cos\left(\omega t\right)+N\left(t\right)$$

where β represents the damping factor, A means the periodic signal amplitude, ω represents the driving frequency, U(x) stands for the potential function model. The hidden function model in this paper was inspired by the literature [35], whose expressions are, where b are the system coefficients (the coefficient values are greater than 0), $N\left(t\right)=\sqrt{2D}\xi \left(t\right)$ represents the noise term, whereas ξ(t) is the additive Gaussian white noise with zero mean, and satisfies

(31) 〈N(t)N(t+τ)〉=2Dδ(t) 

where D is the noise intensity, δ(t) is the Dicla function, and τ is the time interval.

In this paper, the discrete four-level Runge–Kutta method was used to solve.

(32) $$\{\begin{array}{c} y_{1}=y\left[n\right];\\ y_{2}=y\left[n\right]+x_{1}h/2;\\ y_{3}=y\left[n\right]+x_{2}h/2;\\ \begin{array}{c} y_{4}=y\left[n\right]+x_{3}h;\\ x_{1}=-U^{\prime }\left(x\left[n\right]\right)-\gamma y_{1}+S\left[n\right]+N\left[n\right];\\ x_{2}=-U^{\prime }\left(x\left[n\right]+y_{1}h/2\right)-\gamma y_{2}+S\left[n\right]+N\left[n\right];\\ \begin{array}{c} x_{3}=-U^{\prime }\left(x\left[n\right]+y_{2}h/2\right)-\gamma y_{3}+S\left[n+1\right]+N\left[n+1\right];\\ x_{4}=-U^{\prime }\left(x\left[n\right]+y_{3}h\right)-\gamma y_{4}+S\left[n+1\right]+N\left[n+1\right];\\ x\left[n+1\right]=x\left[n\right]+\left(y_{1}+2y_{2}+2y_{3}+y_{4}\right)h/6;\\ y\left[n+1\right]=y\left[n\right]+\left(x_{1}+2x_{2}+2x_{3}+x_{4}\right)h/6; \end{array} \end{array} \end{array}$$

**Figure 1:**
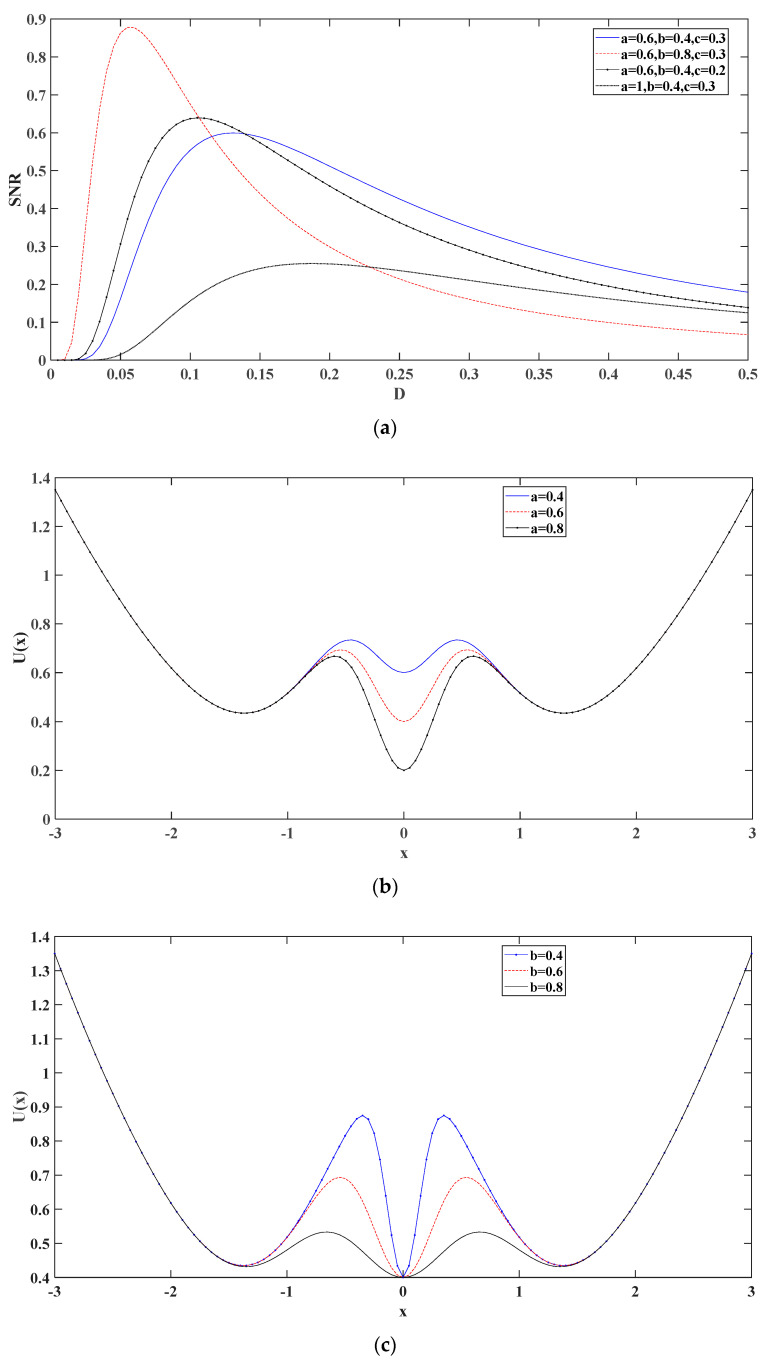

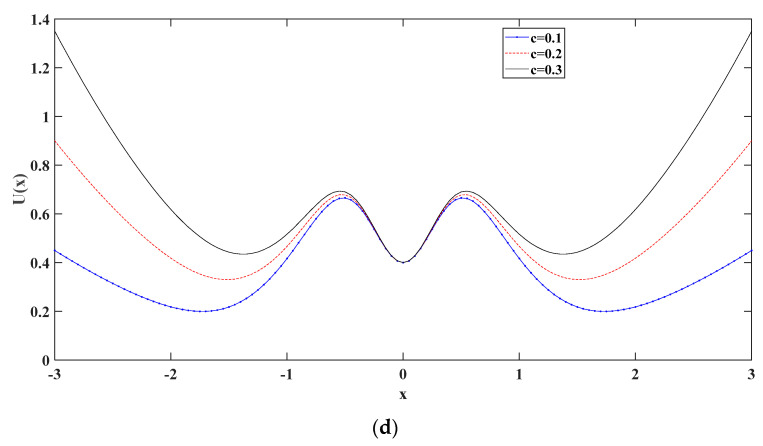
Comparison of the output signal-to-noise proportion and potential function under different coefficients of the approach put forward ((**a**–**d**) are the effects of different parameters on the potential function, respectively).

Figure 1 compares the output signal-to-noise proportion and potential function for different parameters, from which it can be seen that the parameters greatly influence the output of the stochastic resonance model. The red curve is high because the trap walls of the two steady states on the side and the steady middle state are not so steep after b becomes larger, which is favorable to the resonance situation when the noise is low. However, as the noise increases, the noise amplification effect of SR gradually decreases. The black point curve (the weakest one of SNR) is because a becomes larger, the depth of the intermediate steady-state trap is too deep, and the trap wall is too steep, thus making it more difficult for the particle to jump out of the current steady-state at the intermediate steady-state. As such, it is not beneficial for the occurrence of random resonance. Hence, to address this problem, the proposed method was further improved in this paper using an adaptive algorithm to find the optimal parameters to achieve the best output for extracting the eigenfrequencies.

### 3.2. Improved Moth Flame Optimization Algorithm

From the bionic principle of MFO, it can be observed that the convergence ability, the optimization-seeking accuracy, and the ability to exploit the unknown domain of the algorithm are mainly influenced by its flight mode and evolutionary mechanism. Moth flame optimization algorithm has the merits of quick convergence speed and simple structure. However, the algorithm is more likely to divide into local optimal solutions for some complex optimization problems. This is especially true when optimizing high-dimensional problems or multi-peaked functions, in which the phenomena of falling into local optimums will be more obvious. The optimization ability of the original moth flame optimization algorithm depends mainly on the interactions between individuals. When a single individual falls into a local optimum, it can escape through the flame position. However, if most search individuals reach the local optimum, the search process of the whole algorithm will slow down and eventually stall. An enhanced moth flame improvement algorithm was put forward in this paper to prevent moth individuals from falling into local minima in the search and help the algorithm converge to the best solution quickly.

Levy flight was first used to obtain a larger search space, considering that the flight mode of the moth has a large influence on the ability of the algorithm to exploit the unknown domain. In the 1930s, the French mathematician Levy proposed the Levy flight strategy to explore a more ideal way to search for food for organisms in an unknown environment [36]. Levy flight can be simply described as a moving entity that moves most of the time over very short distances and occasionally performs an unusually large step motion. Adding Levy flight to the MFO algorithm can expand the algorithm. Levy flight is one of the best representations of the random wandering model.

The position update formula for Levy’s flight is shown in Equation (33)

(33) $$x_{i}^{t+1}=x_{i}^{t}+\alpha ⊗Levy$$

where $Levy=\frac{u}{{\left|v\right|}^{\frac{1}{\lambda }}}$ is λ generally taken as (1,3], v~N(0,1) denotes the step control variable, and ⊗ denotes the point-to-point multiplication.

Then, considering that the MFO algorithm tends to fall into local optimality when optimizing high-dimensional problems or multi-peaked functions, this paper improved the location update by using the gradient method in the GBO method [37]. The approach put forward in this paper was inspired by the GBO approach put forward in [37] and led to the coyote optimization algorithm. As an optimization algorithm, the GBO approach integrates the gradient approach and the population approach and is made up of two major parts. One of them is GSR on the basis of the gradient search (GB) approach. In the gradient search rule (GSR), a better position in the FEASIBLE area is searched and obtained b controlling the motion of the vector so as to enhance the trend of exploration and boost the convergence of GBO. Nevertheless, the extraction of rules from Newton’s gradient-based method was made [38]. On the basis of the reality that there are a lot of non-differentiable optimization problems, the numerical gradient approach was applied in this work rather than the direct function derivation approach. Nevertheless, one sample at a time for gradient descent is used by the numerical gradient approach. Hence, for precision, only one sample per training is adopted by the stochastic random gradient descent approach so as to decide the direction of the gradient. While acquiring local minima, high accuracy rate can be obtained. For convergence rate, one sample is iterated by the stochastic gradient descent approach at a time. Hence, the iteration direction differs significantly and does not converge fast to the optimal local solution. Hence, this paper proposed that the positive cosine algorithm [39] replaces the GSR algorithm in the GBO approach to enhance the overall convergence rate.

### 3.3. Weak Signal Detection Strategy Based on ATSR

The key theory of adaptive methods is to dig the best value of the objective function [40]. The merits and demerits of adaptive approaches are determined by the objective function directly. The signal-to-noise proportion is the most frequently adopted optimization objective in adaptive SR approaches [41]. Hu and Li [24] observed that combining the highest spectral peak and signal-to-noise proportion was more reasonable than the traditional signal-to-noise ratio optimization objective. In this method, if the frequency of the input signal cannot be accurately estimated, the maximum value in the output spectrum is used rather than the frequency amplitude of the input signal to calculate the signal-to-noise proportion of the SR output. This method was also used by the authors of [24]. We considered the problem of excessive noise intensity of weak magnetic signals and used improved sample entropy [42] as one of the parameters to evaluate the output results. The specific implementation of the method was as below

**Figure 2:**
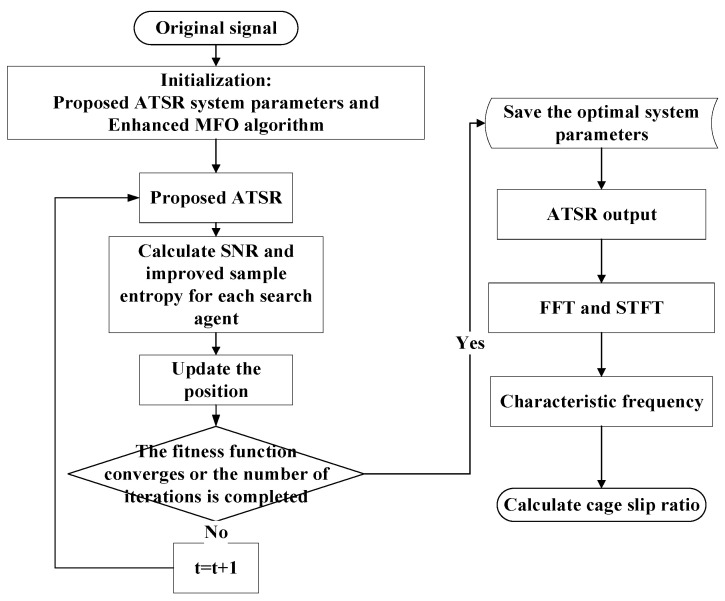
Flow chart of the proposed ATSR method for weak magnetic signals.

(1) Input the analysis signal and assign the ATSR system parameters to search range and Enhanced MFO algorithm parameters. The number of nests should be selected according to the specific situation: in the case of larger number of nests, the accuracy of the solution will be better, but the speed and efficiency of the solution will decrease; the smaller the number of nests, the greater the speed and efficiency of the solution, but the accuracy of the solution will worsen. Unless noted, the search range, most number of iterations, and number of search agents of the stochastic resonance system were set to [0,30] and 30, respectively.
(2) Calculate the ATSR output signal. It is worth noting that the classical SR is only suitable for small parameter signals, i.e., f≪1 (f means the signal frequency). Hence, if the input signal cannot meet the application requirements of classical SR, a large-signal proportional transformation will be used. This was done in the current work in order to accommodate the ATSR [43] method proposed.
(3) The minimum value is found with the cuckoo search algorithm, while the maximization of the signal-to-noise proportion of the output signal is the metric of the detection effect of the weak magnetic signal in the proposed method. Therefore, to reduce the interference of noise on the signal results, we selected the improved sample entropy [42] as the objective function, normalized the two, and took the reciprocal of its normalized result as the seeking target of the cuckoo search algorithm. For each search agent, the signal-to-noise proportion of the SR output signal was calculated on basis of the literature [24].
(4) Update the location of the search.
(5) Decide if the termination condition was reached, i.e., if $t\geq T_{\max}$ (t means the present iteration). If yes, end the iteration. Otherwise, make t=t+1 and keep the iteration. In this paper, we set the termination condition as the state of having reached search accuracy or the maximum number of iterations.
(6) Get and save the optimal coefficients. Then, obtain the best ATSR output with the optimal coefficients.
(7) Analyze the optimal output signal with FFT and STFT, and extract the characteristic frequencies on the basis of the highest peak of the FFT spectrum. Using STFT, extract the signal characteristic frequency for the variable operating conditions. The flow of the ATSR approach put forward is displayed in Figure 2.

**Figure 3:**
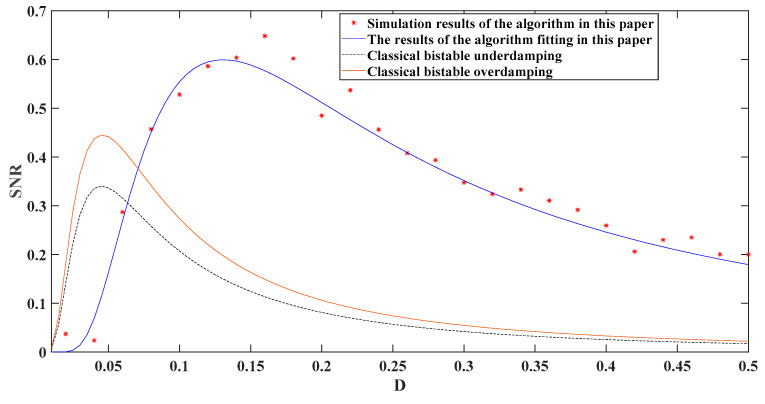
Comparison of theoretical simulation results of different methods.

Through theoretical simulation, different methods were compared with the ATSR approach put forward in this paper, and the outcomes are displayed in Figure 3. From Figure 3, the method put forward in this paper effectively improved the output signal-to-noise proportion

## 4. Results

### 4.1. Numerical Analysis

**Figure 4:**
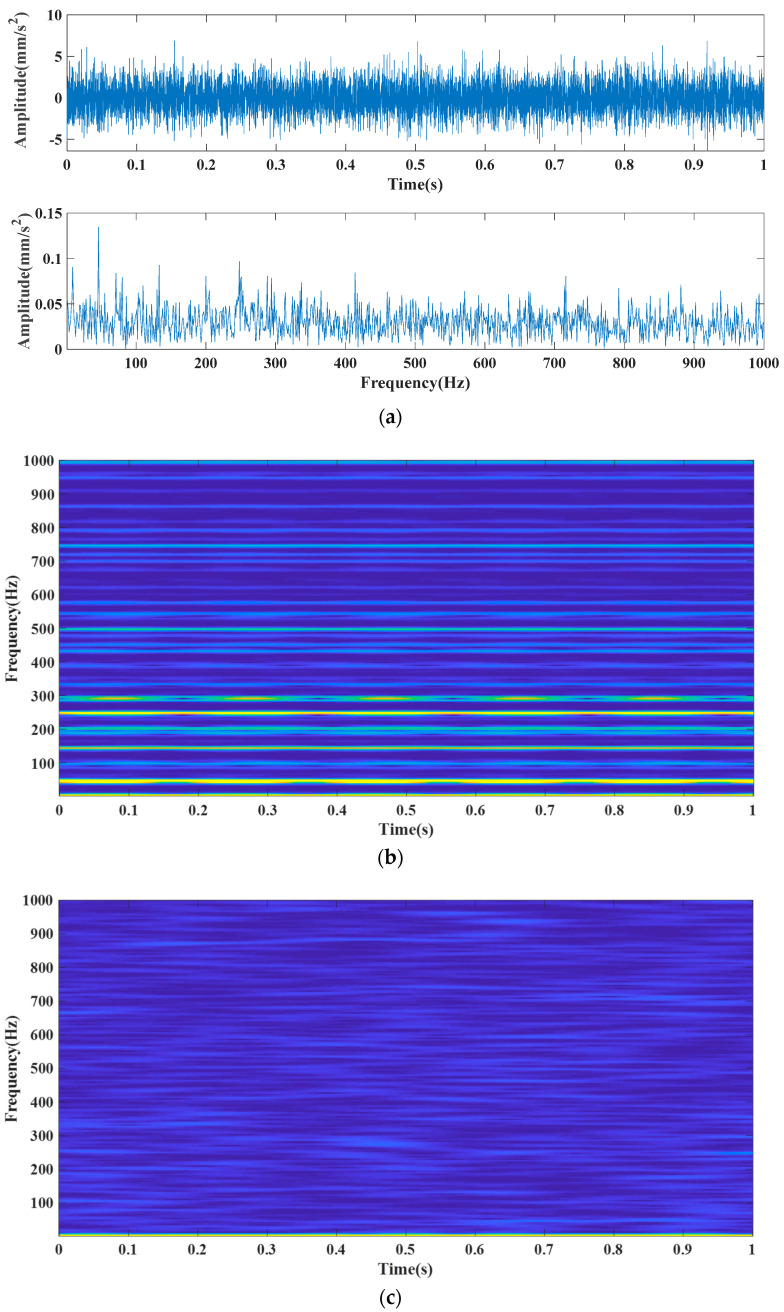
Time-domain and envelope spectra of the simulated signal, where (**a**) is the signal time domain and envelope spectrum; (**b**) is the time and frequency diagram of the noise-free signal; (**c**) is the time and frequency diagram of the noise-added signal.

For verifying the effectiveness of the approach put forward in this paper for weak magnetic signals, a strong noise simulation signal containing three characteristic frequencies was used. Two types of white noise and increased noise intensity were added to the noise section to emphasize the characteristics of the weak signal. In the next section, experimental signals from engineering simulation tests were adopted to examine the approach put forward. The combination of simulated and experimental signals profoundly validated the merits of the approach put forward in rolling bearing weak magnetic signal processing. The simulated signal is shown in Equation (34) [39].

(34) $$\{\begin{array}{c} x\left(t\right)=s\left(t\right)+n\left(t\right)=\sum_{i}A_{i}h\left(t-iT-\tau_{i}\right)+n\left(t\right)\\ A_{i}=A_{0}\sin\left(2\pi f_{r}t+\varphi_{A}\right)+C_{A}\\ h\left(t\right)=e^{-Ct}\cos\left(2\pi f_{n}t+\varphi_{m}\right) \end{array}$$

where s(t) means the periodic error shock; $f_{r}$ stands for the frequency conversion, and $f_{r}=46\;\mathrm{Hz}$; $C_{A}$ represents 1; C means the attenuation coefficient (C=760); $f_{n}$ stands for the resonance frequency ($f_{n}=3000\;\mathrm{Hz}$), and $f_{i}$ means the feature frequency of the inner ring error ($f_{i}=1/T=145,249\mathrm{Hz}$). $\tau_{i}$ represents the slight sliding of the i−th shock concerning period T, after which a normal distribution with a mean of 0 (standard deviation is 0.5% is the Gaussian white noise with an average of 0.5% of the frequency conversion) is followed by the sliding. n(t) means Gaussian white noise with an SNR of −15 dB, and $f_{s}$ means the sampling frequency ($f_{s}=8192\;\mathrm{Hz}$). The number of analysis data points was 8192 according to Figure 4. The reason for applying the synthetic signal is that the analog signal in Equation (34) allows two characteristic frequencies to appear in one signal simultaneously, simulating the bearing in the case of a simultaneous failure of the inner and outer ring. This provided a clear and intuitive response to the superiority of the method proposed in this paper; the synthetic signal was chosen to verify our method’s extraction capability in a strong, noisy, multi-feature frequency signal. Figure 4 shows the time-domain and envelope spectra of the simulated signal used in this paper. For verifying the superiority of the proposed optimization algorithm, the underdamped tri-stable stochastic resonance system model without improved MFO optimization was added to this paper for comparative analysis.

**Figure 5:**
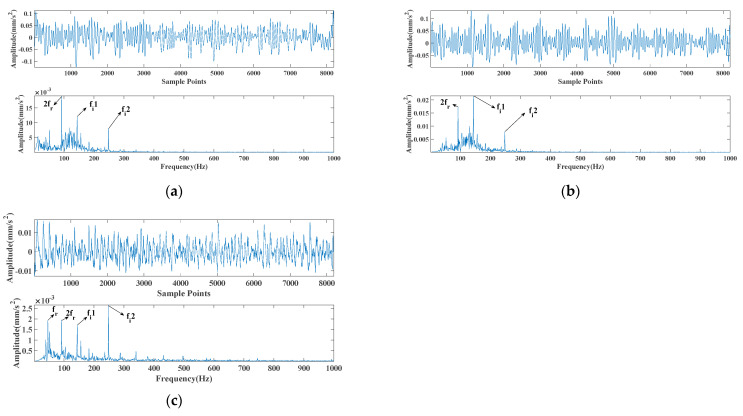
Time domain and envelope spectra after filtering for three methods: (**a**) traditional SR method; (**b**) MFO optimized ATSR method; (**c**) improved MFO optimized ATSR method.

**Figure 6:**
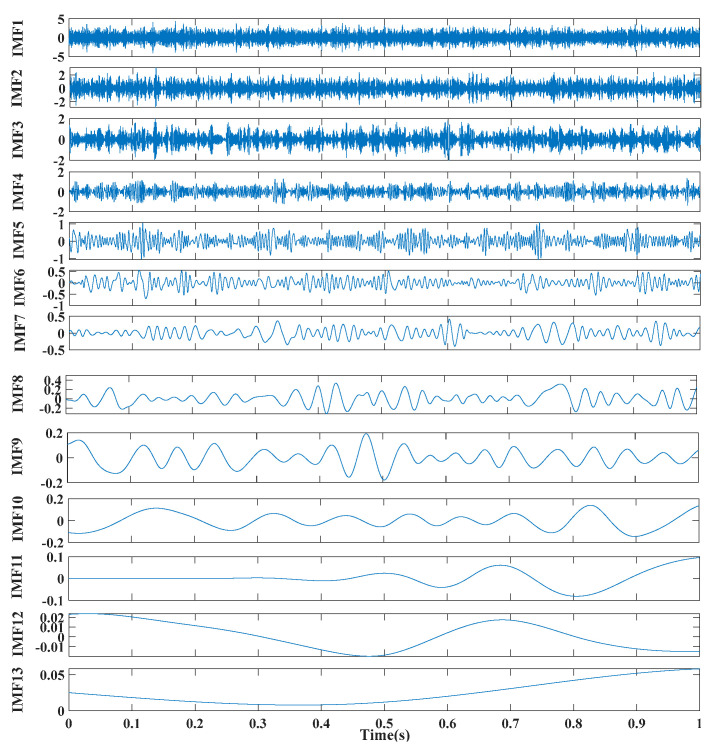
CEEMDAN decomposition results.

**Figure 7:**
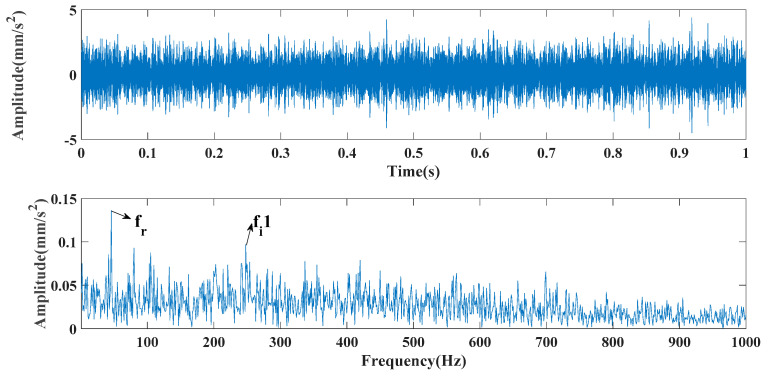
CEEMDAN decomposition result envelope spectra.

**Figure 8:**
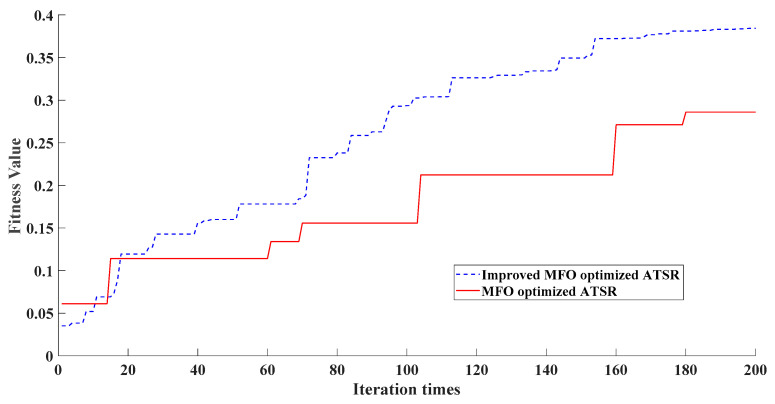
Improved MFO versus MFO optimized adaptation values.

Figure 5, Figure 6 and Figure 7 show the time domain and envelope spectra after filtering by different algorithms, respectively. From Figure 5, it can be seen that the proposed method effectively extracted the transient frequency and two characteristic fault frequencies. In contrast, the traditional method and the unimproved MFO method were affected by the parameters—or the optimization algorithm needed to be further improved—leading to inaccurate signal and still noise interference. Figure 8 displays the improvement of the MFO approach before and after the improvement of the optimization efficiency. To quantify and compare the results of different methods, Table 1 shows the input signal-to-noise ratio, output signal-to-noise ratio, sample entropy, and correlation coefficient for comparison.

The approach put forward in this paper exhibited good practicality for reprocessing weak multi-featured frequency signal species and could be applied to engineering practice.

### 4.2. Engineering Experimental Platform Analysis

**Figure 9:**
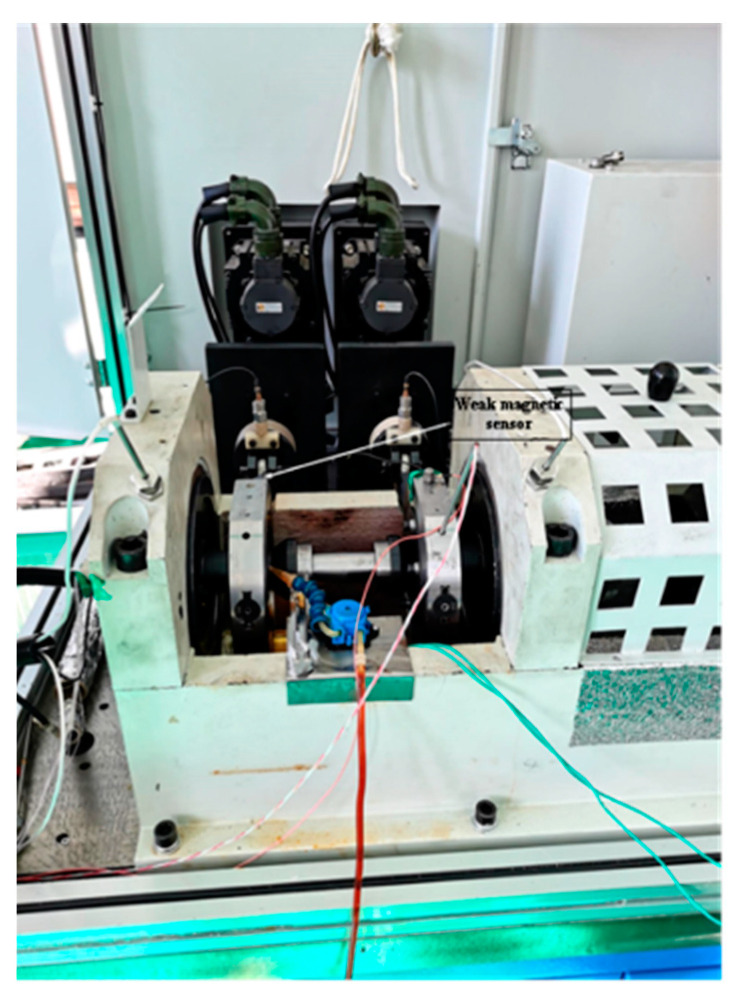
Physical diagram of the experiment table.

The test platform included the test platform and the measurement system. The test platform part consisted of the test bench and the control system. Figure 9 displays the physical diagram of the test bench, which consisted of the test bearing, test spindle, accompanying test bearing, driving unit, bearing outer ring fixture, and loading system. The test was fallen into two sets of bearings under test and two sets of companion bearings. Since this paper was aimed at weak magnetic signal detection, only the weak magnetic signal sensor is labeled in the figure. Since the bearings used in this study were nonmediated, the outer ring of the bearing was not involved in the motion, and the drive shaft only made the inner ring, rolling body, and cage rotate. Furthermore, the cage was made of nonpermeable material, so there was no signal component of the cage and outer ring in the weak magnetic signal sensor, only the signal component of the rolling body and inner ring. At the same time, the permeability of the inner ring was much larger than that of the rolling body, which resulted in a much smaller amplitude of the detected rolling body signal than that of the examined inner ring signal. The signal of the rolling body modulated the detected signal of the inner ring, therefore, the collected signals needed to be processed using the approach put forward in this paper to extract the rotational feature frequencies of the rolling body and the inner ring. The bearing parameters used in this case are shown in Table 2.

The experimental design of this paper was as follows: To verify that the approach put forward in this paper was practical and feasible, we designed a variable speed experiment. The speeds were: 200 rpm/min, 900 rpm/min, 1600 rpm/min, 3200 rpm/min and 4800 rpm/min, loaded with: 100 N, 200 N, 200 N, 200 N, 200 N, and 200 N, respectively. Meanwhile, the experiments in this paper were conducted without adding lubricant. The sampling rate of this signal was 30 kHz.

The cage slip rate was obtained from the ratio of kinematics to the detected holding and rotational speed [4].

(35) $$S_{c}=1-\frac{v_{m}}{v_{c}}$$

where $v_{m}$ represents the monitored cage speed and $v_{c}$ represents the theoretical speed of the cage in the case of absolute rotation. Meanwhile, Equation (35) can be expressed in terms of the characteristic frequency

(36) $$S_{c}=1-\frac{f_{m}}{f_{c}}$$

where $f_{m}$ is the cage rotation frequency seen and $f_{c}$ is the theoretical rotation frequency of the cage. The literature [5] states that the theoretical rotation speed of the cage can be calculated from the rotational speed of the inner ring.

(37) $$f_{c}=\frac{f_{r}}{2}\left(1-\frac{d\cos\alpha }{D}\right)$$

where, $f_{r}$ is the rotation frequency of the inner ring, d the diameter of the rolling body, is the D diameter of the pitch circle, and α is the contact angle.

Substituting Equation (37) into Equation (36), so get

(38) $$S_{c}=1-\frac{f_{m}}{f_{r}\left(\frac{1}{2}-\frac{d\cos\alpha }{2D}\right)}$$

From Equation (38), the cage slippage rate can be calculated by the cage rotation frequency and the inner ring rotation frequency. At the same time, the rotation frequency of the cage and the rotation frequency of the rolling body have the following relationship:

(39) $$f_{m}=\frac{f_{ro}}{Z}$$

where $f_{ro}$ denotes the rotation frequency of the rolling body and Z denotes the number of rolling bodies. Therefore, the final cage slip rate can be obtained by associating Equation (38) with Equation (39) as

(40) $$S_{c}=1-\frac{f_{ro}}{f_{r}\left(\frac{1}{2}-\frac{d\cos\alpha }{2D}\right)Z}$$

**Figure 10:**
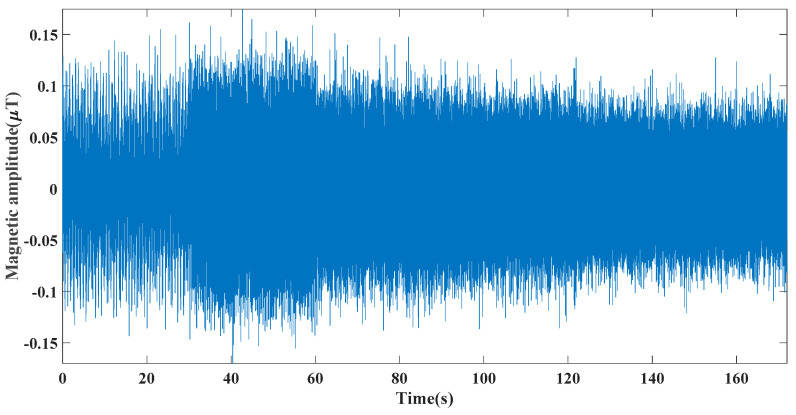
Weak magnetic signal time domain.

**Figure 11:**
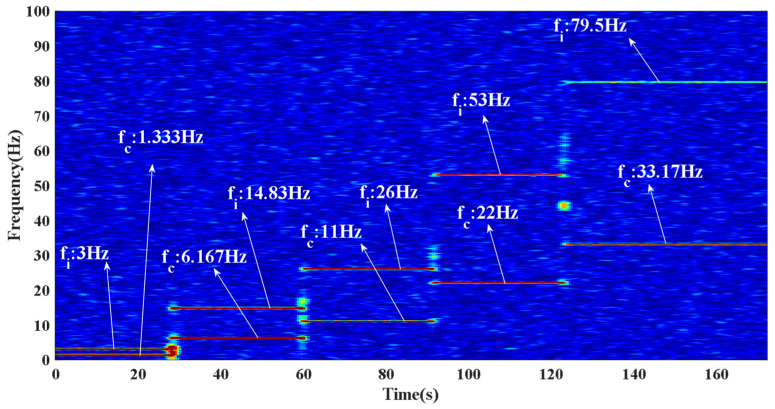
Time and frequency domain of weak magnetic signal after noise reduction.

Based on the above equations and bearing parameters, the theoretical speeds of the bearing cage in this experiment were calculated as 1.40 Hz, 6.28 Hz, 11.16 Hz, 22.32 Hz, and 33.48 Hz. Figure 10 displays the time domain plots of the signals gathered in this experiment. Figure 11 displays the STFT results after the feature extraction with the approach put forward in this paper. The STFT is chosen instead of the envelope spectrum because the experiment in this paper is a variable speed experiment, and the time-frequency plot can visualize the experimental process. As shown in Figure 11, after filtering by the approach put forward in this paper, the feature frequencies of the cage and inner ring rotational speeds can be seen. The comparison between the theoretically calculated values and the actual measured values in Table 3 shows that only weak sliding occurs in this experimental bearing at low speed and light load, and the reason considered is that no lubricant is added in this experiment. Experiments prove that the approach put forward can efficiently filter the weak magnetic signal and extract feature frequencies, which has high practicality.

## 5. Conclusions

This paper addressed the problems of strong and weak magnetic signal noise and difficulty in extracting weak characteristic frequencies when monitoring bearing slip utilizing weak magnetic detection. It further proposed a method for adaptively optimizing the parameters of the underdamped tri-stable stochastic resonance system model with an improved MFO so that it could accomplish adaptive adjustment according to different signals. Numerical simulations and case studies showed that the method adapted to optimal parameters according to the best signal-to-noise ratio, solving the drawback of artificially set parameters. We concluded that the method outperformed the conventional fixed-parameter SR approach, MFO-SR approach, and the CEEMDAN approach in detecting bearing slippage of weak magnetic signals. Therefore, this work solved the problem of processing weak magnetic signals and has provided ideas for subsequent research on signal processing. Future work could address the extended application of weak magnetic detection means and the effects of different noises on the processing of stochastic resonance systems in weak magnetic signals.

## Figures and Tables

**Table 1:** Comparison of quantitative indicators of methods.

Method	Input Signal to Noise Ratio (dB)	Output Signal to Noise Ratio (dB)	Sample Entropy	Correlation Coefficient
Improved MFO Optimized ATSR	−15	4.37	1.22	0.86
MFO Optimization Improvement SR	−15	4.11	1.24	0.85
Traditional SR	−15	2.65	1.48	0.76
CEEMDAN	−15	2.43	1.60	0.74

**Table 2:** Bearing coefficients.

Ball Number N	Pitch Diameter D	Roller Diameter d	Contact Angle α
14	46	7.5	0

**Table 3:** Comparison of theoretical and actual values at different speeds.

Inner Ring Speed (rpm)	Inner Ring Theoretical Rotation Frequency (Hz)	Measurement RPM (Hz)	Theoretical Rotation Frequency of Cage (Hz)	Actual Rotation Frequency of Cage (Hz)
200	3.33	3	1.40	1.333
900	15	14.83	6.28	6.167
1600	26.67	26	11.16	11
3200	53.33	53	22.32	22
4800	80	79.5	33.48	33.17

## Data Availability

Not applicable.
